# SPECT/CT Imaging of High-Risk Atherosclerotic Plaques using Integrin-Binding RGD Dimer Peptides

**DOI:** 10.1038/srep11752

**Published:** 2015-06-30

**Authors:** Jung Sun Yoo, Jonghwan Lee, Jae Ho Jung, Byung Seok Moon, Soonhag Kim, Byung Chul Lee, Sang Eun Kim

**Affiliations:** 1 Department of Nuclear Medicine, Seoul National University Bundang Hospital, Seoul National University College of Medicine, Seongnam, Republic of Korea; 2 Smart Humanity Convergence Center, Program in Biomedical Radiation Sciences, Department of Transdisciplinary Studies, Graduate School of Convergence Science and Technology, Seoul National University, Suwon 443-270, Republic of Korea; 3 Center for Nanomolecular Imaging and Innovative Drug Development, Advanced Institutes of Convergence Technology, Suwon 443-270, Republic of Korea; 4 Institute for Bio-Medical Convergence, College of Medicine, Catholic Kwandong University, Gangneung 210-701, Republic of Korea; 5 Catholic Kwandong University International St. Mary’s Hospital, Incheon 404-834, Republic of Korea

## Abstract

Vulnerable atherosclerotic plaques with unique biological signatures are responsible for most major cardiovascular events including acute myocardial infarction and stroke. However, current clinical diagnostic approaches for atherosclerosis focus on anatomical measurements such as the degree of luminal stenosis and wall thickness. An abundance of neovessels with elevated expression of integrin α_v_β_3_ is closely associated with an increased risk of plaque rupture. Herein we evaluated the potential of an α_v_β_3_ integrin-targeting radiotracer, ^99m^Tc-IDA-D-[c(RGDfK)]_2_, for SPECT/CT imaging of high-risk plaque in murine atherosclerosis models. *In vivo* uptake of ^99m^Tc-IDA-D-[c(RGDfK)]_2_ was significantly higher in atherosclerotic aortas than in relatively normal aortas. Comparison with the negative-control peptide, ^99m^Tc-IDA-D-[c(RADfK)]_2_, proved specific binding of ^99m^Tc-IDA-D-[c(RGDfK)]_2_ for plaque lesions in *in vivo* SPECT/CT and *ex vivo* autoradiographic imaging. Histopathological characterization revealed that a prominent SPECT signal of ^99m^Tc-IDA-D-[c(RGDfK)]_2_ corresponded to the presence of high-risk plaques with a large necrotic core, a thin fibrous cap, and vibrant neoangiogenic events. Notably, the RGD dimer based ^99m^Tc-IDA-D-[c(RGDfK)]_2_ showed better imaging performance in comparison with the common monomeric RGD peptide probe ^123^I-c(RGDyV) and fluorescence tissue assay corroborated this. Our preclinical data demonstrated that ^99m^Tc-IDA-D-[c(RGDfK)]_2_ SPECT/CT is a sensitive tool to noninvasively gauge atherosclerosis beyond vascular anatomy by assessing culprit plaque neovascularization.

Cardiovascular diseases are the number one cause of morbidity and mortality worldwide^1^. By 2030, they are projected to be the single leading cause of death globally^2^. Rupture of atherosclerotic plaques (i.e., atheromatous plaques in the inner lining of the arteries) is a critical feature leading to major clinical events such as acute myocardial infarction, sudden cardiac death, and stroke. Monitoring plaque progression and identifying vulnerability may help prevent these events and reduce the burden of cardiovascular disease^3^. At present, clinicians lack reliable tools to detect high-risk plaques that are rupture-prone and to predict the location of possible adverse events^3^,^4^. Current diagnostic strategies predominantly focus on anatomical issues such as myocardial ischemia^5^, hemodynamic luminal narrowing, or morphological abnormalities of atheromas, but not on the biological aspects of atherosclerotic lesions. This traditional strategy has proven disappointing in preventing myocardial infarction or prolonging life, except in limited patient groups^6^,^7^,^8^.

Atherosclerotic plaques comprise a heterogeneous mixture of cellular and acellular elements^9^. In the past decade, considerable efforts have been devoted to determine the specific compositional features of unstable vulnerable plaques^9^,^10^,^11^,^12^. Atherosclerotic plaques at the highest risk of rupture clearly exhibit a large lipid-rich necrotic core, thin fibrous cap, neovascularization, spotty calcium, and abundant inflammatory cells; these features are distinctive from those of stable lesions^9^,^10^,^11^. Therefore, the assessment of plaque composition is potentially more important than the traditional detection of simple intraluminal stenosis for predicting devastating arterial events^7^,^8^,^13^,^14^. As a result, there is a compelling need to develop diagnostic imaging techniques to gauge the biological details of plaques that trigger the conversion of asymptomatic atheromas to rupture-prone lesions and subsequent fatal thrombosis. Molecular imaging strategies now provide an approach other than assessing vessel stenosis and wall thickness, and shed light on the *in vivo* pathology of atherosclerotic plaques^13^,^14^,^15^,^16^.

In particular, neovascularization is a key process of advanced atherosclerotic plaques and an independent predictor of future adverse clinical outcomes^17^,^18^,^19^. Newly formed vasculature within a plaque is closely related to the inflammatory process, which is another important determinant of plaque rupture because it facilitates monocyte recruitment and transmigration. Intraplaque hemorrhage, a critical event that provokes lesion destabilization by providing erythrocyte-derived phospholipids and free cholesterol, is caused by damage to neovessels because of their immature, fragile characteristics resulting from the lack of smooth muscle cells^20^. Aspects of neoangiogenesis have therefore surfaced as emerging major targets for molecular imaging of atherosclerosis. Angiogenesis has been extensively studied for cancer diagnosis^21^; however, imaging neovasculature to identify patients at risk for major clinical manifestations of atherosclerosis is relatively new, compared to the long history of imaging plaque inflammation^22^,^23^,^24^. Previous investigations of imaging neovascular proliferation in plaques are limited to trials using nanoparticle-enhanced molecular magnetic resonance imaging (MRI)^25^,^26^ and to a few recent studies using positron emission tomography (PET) with simple arginyl-glycyl-aspartic acid (RGD) monomer peptides^27^,^28^.

The RGD, an excellent targeting moiety for integrin α_v_β_3_ of activated endothelial cells, has been successfully validated for imaging tumor angiogenesis in numerous preclinical and clinical studies^21^,^29^,^30^. We recently developed a new radiotracer, ^99m^Tc-labeled RGD peptide (^99m^Tc-IDA-D-[c(RGDfK)]_2_) and performed single photon emission computed tomography (SPECT) imaging for targeting glioblastoma^31^. Compared to other reported RGD monomer-based agents^30^,^32^,^33^ [e.g., ^99m^Tc-(CO)_3_-pyrazoyl conjugate of c(RGDyK)^34^ and ^123^I-c(RGDyV)], the developed RGD dimer agent ^99m^Tc-IDA-D-[c(RGDfK)]_2_ showed specific integrin-binding affinity, high tumor accumulation and desirable pharmacokinetic properties for tumor xenograft imaging^31^. Similar to tumor angiogenesis imaging, this radiotracer is expected to be effective for imaging neovessel-rich atherosclerotic plaques and show better imaging performance compared with previously reported monomeric RGD probe based approaches^27^,^28^.

Here we describe a molecular imaging strategy that uses α_v_β_3_ integrin-targeted probe ^99m^Tc-IDA-D-[c(RGDfK)]_2_ with SPECT to achieve improved atherosclerosis staging through assessment of neovascularization. To illustrate the utility of this approach, we demonstrate SPECT/CT imaging of atherosclerotic mouse models and analyze correlation between *in vivo* uptake of the radiotracer and *ex vivo* autoradiography signal and corresponding histopathological signatures. Comparative measurements with conventional monomeric RGD derivatives reveal superior sensitivity of the designed dimer RGD probe for noninvasive nuclear imaging as well as tissue fluorescence imaging. Finally we discuss the potential of angiogenesis targeted approach for ideal noninvasive imaging to pinpoint high-risk atherosclerotic plaques before they lead to fatal clinical events.

## Results

### Development of High-risk Atherosclerosis and Validation Studies

**Figure 1:**
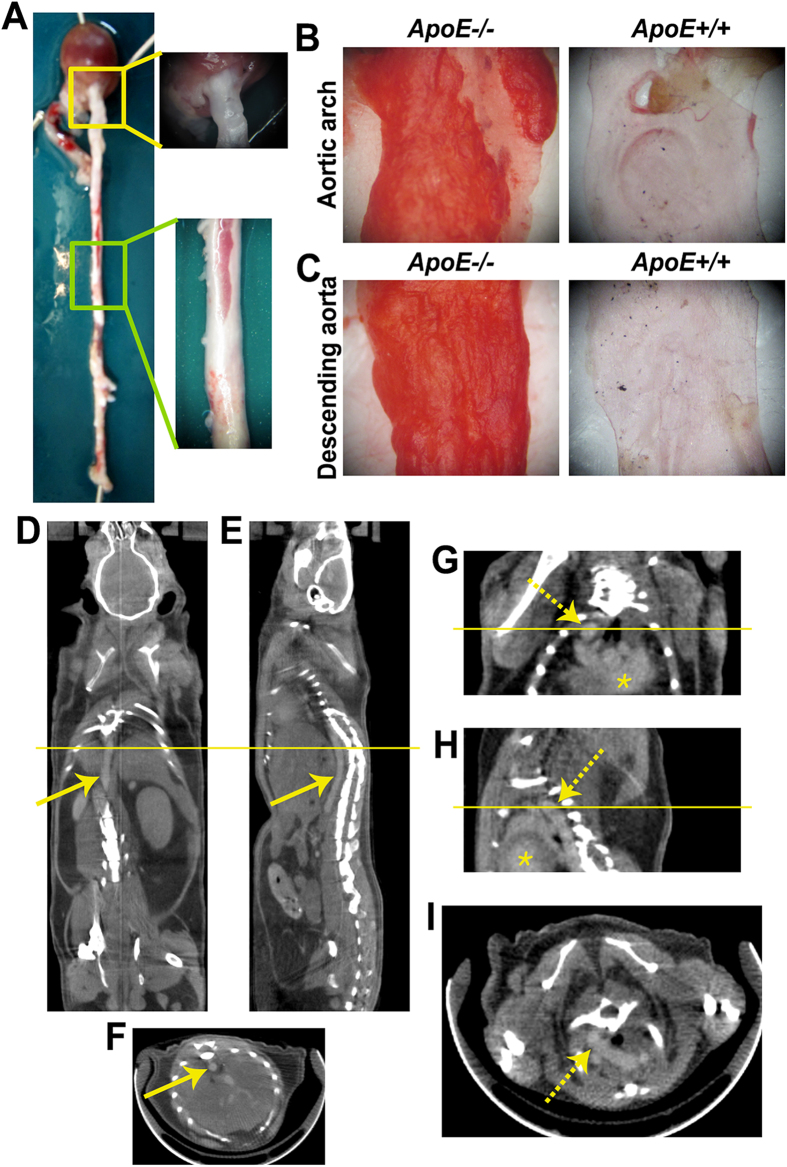
Validation of the atherosclerosis mouse model and structural identification using computed tomography (CT) images. **A,** Representative photographs of an excised heart and aorta with the normal areas filled with blood (red) and the atherosclerotic lesions appearing as white. The regions of the aortic arch (yellow rectangle) and descending aorta (green rectangle) are displayed in high magnification. **B,** Oil-Red-O (ORO) staining of the excised aortic arch tissues and, **C,** the descending aortas in (left) the apoE-deficient mice and (right) the control mice. **D–I**, Representative CT images of atherosclerotic mice at 10 min post-contrast injection (**D** and **G**, coronal views; **E** and **H**, sagittal views; **F** and **I**, transverse views at yellow lines). The arrows denote the descending aortas and the dotted arrows denote the aortic arch regions. The asterisks highlight the heart. ApoE, apolipoprotein E.

To explore the ability of α_v_β_3_ integrin-targeted probes to detect atherosclerotic lesions with a high risk of rupture, we established mouse models of atherosclerosis by feeding a high-cholesterol diet to apoE-deficient (*ApoE-/-*) mice. After 40 weeks of this special diet, their hearts and aortas were excised and carefully analyzed. As Fig. 1A shows, atherosclerotic lesions (white areas) were present in the aorta—predominantly in the aortic arch; in the origins of the brachiocephalic, left subclavian, and left common carotid arteries; and throughout the thoracic and abdominal aorta. After the gross anatomical examination, we performed Oil-Red-O (ORO) staining of the excised aortic arch tissues and descending aortas in the apoE transgenic mice and the wild-type C57BL/6J mice (Figs 1B,C). Lipid pool zones with a strong ORO-positive response were identified in the apoE-deficient mice, compared to the control mice, which demonstrated significant development of lipid-rich high-risk lesions; this was observed in a previous study^35^.

The x-ray CT scans with a vascular contrast agent revealed detailed anatomy of heart and aorta regions, which included the aortic arch and descending aorta structures (Figs 1D–I). As a complement to SPECT imaging, x-ray CT provides structural high-resolution visualization of specific locations for developed atherosclerotic lesions.

### ^9m^Tc-IDA-D-[c(RGDfK)]_2_ Imaging of Vulnerable Atherosclerotic Plaques

**Figure 2:**
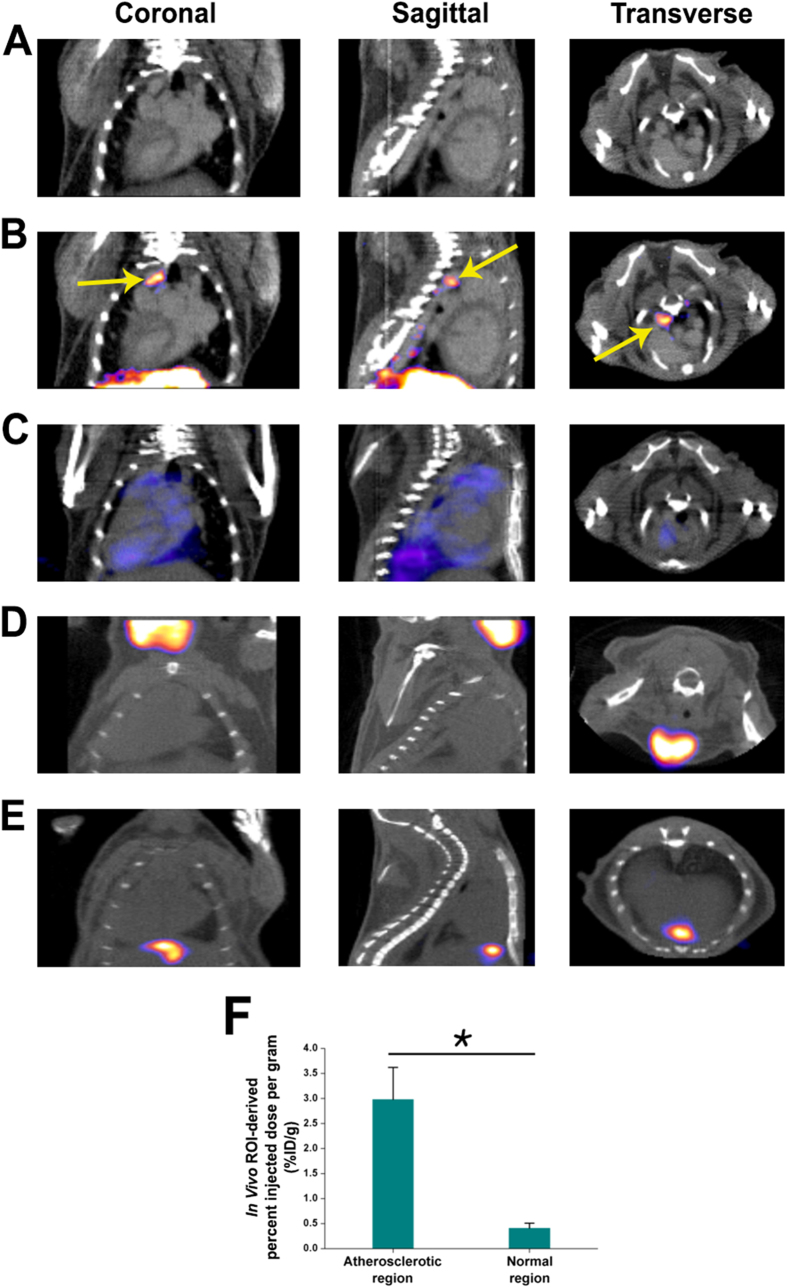
*In vivo* single photon emission computed tomography/computed tomography (SPECT/CT) imaging of high-risk atherosclerotic plaques by using ^99m^Tc-IDA-D-[c(RGDfK)]_2_. Representative coronal, sagittal and transverse planar CT images of an atherosclerotic mouse at 10 min post-contrast injection (**A**) and SPECT-CT fusion images of an atherosclerotic mouse after the injection of (**B**) ^99m^Tc-IDA-D-[c(RGDfK)]_2_ or (**C**) the negative-control peptide ^99m^Tc-IDA-D-[c(RADfK)]_2_, respectively. In the same mouse, the aortic arch region (arrows) containing high-risk plaques show strong SPECT signal only by ^99m^Tc-IDA-D-[c(RGDfK)]_2_, but not by ^99m^Tc-IDA-D-[c(RADfK)]_2_. Representative example of an atherosclerotic mouse after the injection of (**D**) ^99m^Tc-pertechnetate. Only nonspecific signal exist in the salivary glands when injected with ^99m^Tc-pertechnetate. **E**, Representative acquisition of ^99m^Tc-IDA-D-[c(RGDfK)]_2_ in a wild-type mouse (C57BL/6J). Nonspecific ^99m^Tc-IDA-D-[c(RGDfK)]_2_ retention is only present in the gall bladder. **F**, Quantitative analysis of *in vivo*^99m^Tc-IDA-D-[c(RGDfK)]_2_ uptake in atherosclerotic and relatively normal aorta regions of atherosclerotic mice. (*P < 0.001). The data are presented by the mean ± the standard deviation of four different animals. The mice were imaged 30 min after the intravenous injection of each probe. The SPECT images were acquired during 30 min at 30 min post-injection of the radiolabeled probe. %ID/g, percentage injected dose per gram of tissue; ROI, region of interest.

Using a similar radiolabeling protocol as in a previous report^31^, we prepared ^99m^Tc-IDA-D-[c(RGDfK)]_2_, a diagnostic imaging agent for angiogenesis with chemical and radiochemical purities greater than 99% and specific activity greater than 55 GBq/μmol. This agent was designed to have enhanced hydrophilicity of the integrin-binding RGD dimer peptide. Its superior pharmacokinetic properties and high metabolic stability have been proven in a previous study^31^. Herein we evaluated the feasibility of SPECT/CT imaging using ^99m^Tc-IDA-D-[c(RGDfK)]_2_ to noninvasively detect high-risk plaques in established mouse models of atherosclerosis by feeding a high-cholesterol diet to *ApoE-/-* mice (Fig. 2). High local signals of ^99m^Tc-IDA-D-[c(RGDfK)]_2_ in the aortic arch lesions (i.e., well-known prevalent plaque regions, which is identified by contrast-enhanced CT scans in Fig. 2A^35^) was detected at 30 min post-injection (Fig. 2B). We next compared the imaging performance by using the same mouse with the ^99m^Tc-labeled negative-control peptide ^99m^Tc-IDA-D-[c(RADfK)]_2_ (Fig. 2C) on the next day. In the aortic arch region containing atherosclerotic plaques, we observed marked uptake by ^99m^Tc-IDA-D-[c(RGDfK)]_2_ but only scant uptake by ^99m^Tc-IDA-D-[c(RADfK)]_2_. The injection of pure radioisotope ^99m^Tc-pertechnetate in a transgenic mouse showed no specific accumulation in the same territories and only nonspecific uptake in the salivary glands (Fig. 2D). An unmanipulated wild-type C57BL/6J control mouse that received ^99m^Tc-IDA-D-[c(RGDfK)]_2_ also showed no significant radioactivity, except noncleared radiotracer signal in the gall bladder (Fig. 2E). Quantification of ^99m^Tc-IDA-D-[c(RGDfK)]_2_ accumulation revealed significantly higher uptake in the atherosclerotic aortas than in the relatively normal thoracic aortas [n = 4, percentage injected dose per gram of tissue (%ID/g) was 2.98 ± 0.64 versus 0.41 ± 0.10, respectively; *P* < 0.001, Fig. 2F]. Preferential *in vivo* accumulation in aortic plaque suggested that ^99m^Tc-IDA-D-[c(RGDfK)]_2_ has good specificity for use in staging high-risk atherosclerosis.

### ^99m^Tc-IDA-D-[c(RGDfK)]_2_ Uptake: Autoradiography and Histopathology

**Figure 3:**
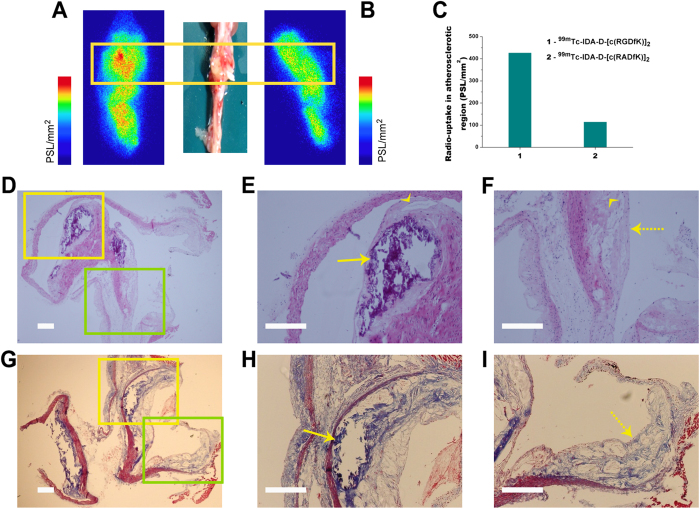
Characterization of ^99m^Tc-IDA-D-[c(RGDfK)]_2_ uptake by autoradiography and histopathology. Representative autoradiography images of (**A**) ^99m^Tc-IDA-D-[c(RGDfK)]_2_ and (**B**) negative-control peptide ^99m^Tc-IDA-D-[c(RADfK)]_2_ of aorta specimens excised from atherosclerotic mice (yellow rectangle) after *in vivo* SPECT/CT imaging. **C**, Results of quantification of the autoradiography image (expressed as photostimulated luminescence per millimeter squared [PSL/mm^2^]); the uptake is compared in the aortic atherosclerosis region for ^99m^Tc-IDA-D-[c(RGDfK)]_2_ and ^99m^Tc-IDA-D-[c(RADfK)]_2_. **D–I**, Histopathological characterization of aortic tissue sections from atherosclerotic animals with high ^99m^Tc-IDA-D-[c(RGDfK)]_2_ uptake. The top and bottom rows show photomicrographs of cross sections of atherosclerotic plaques stained with (**D**–**F**) hematoxylin and eosin and (**G**–**I**) Masson’s trichrome, respectively. **E** and **H**, The high-power views corresponding to the yellow boxes in **D** and **G** indicate the necrotic core (arrows). **F** and **I**, High-power views, which correspond to the green boxes in **D** and **G**, show a fibrous cap (dotted arrows) with the abundance of collagen fibers (blue in **I**). The representative microvessels are denoted by arrowheads. Scale bar, 250 μm.

After *in vivo* SPECT/CT imaging, we further characterized the two radiotracers’ uptake in the aorta of *ApoE-/-* mice by *ex vivo* autoradiography (Figs 3A,B). Autoradiographic uptake in the aortic arch was significantly higher with ^99m^Tc-IDA-D-[c(RGDfK)]_2_ than with the negative-control peptide ^99m^Tc-IDA-D-[c(RADfK)]_2_. This provided good corroboration of the *in vivo* imaging data. Quantitative analysis revealed ^99m^Tc-IDA-D-[c(RGDfK)]_2_ uptake to be 3.7-fold higher than ^99m^Tc-IDA-D-[c(RADfK)]_2_ uptake (Fig. 3C).

We then histopathologically characterized an aorta specimen that showed robust localization of the ^99m^Tc-IDA-D-[c(RGDfK)]_2_ signal by SPECT and by autoradiography (Figs 3D–I). The morphology of rupture-prone atherosclerotic plaques and stable lesions are distinct. Typical signatures of high-risk plaques were present in the specimen: a necrotic core (Figs 3E,H), fibrous cap with an abundance of collagen fibers (Figs 3F,I), and neovascularization (Figs 3E,F), as shown by hematoxylin and eosin staining and Masson’s trichrome staining. Immunofluorescence staining further verified that Integrin α_v_^+^ or Integrin β_3_^+^ activated endothelial cells, CD31^+^ endothelial cells, and CD68^+^ macrophages are enriched in the aortic plaque showing the peptide positive signal (Supplementary Figure 1). Pathological characteristics correlated well with the peak accumulation of ^99m^Tc-IDA-D-[c(RGDfK)]_2_ which suggested that the *in vivo* SPECT signal reflects vulnerable atherosclerotic plaque burden.

### Comparison of ^99m^Tc-IDA-D-[c(RGDfK)]_2_ and ^123^I-c(RGDyV) Imaging

**Figure 4:**
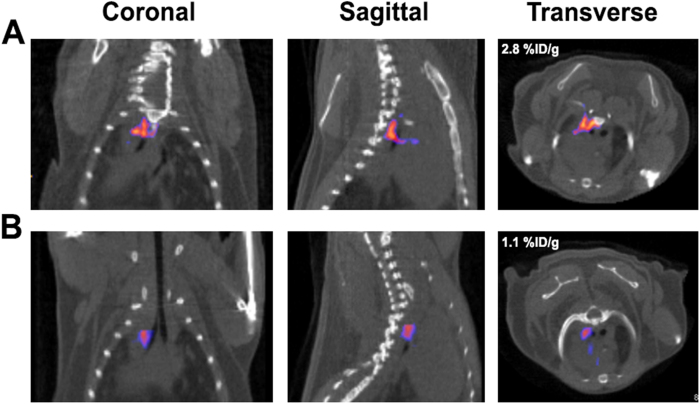
*In vivo* imaging with and quantitative uptake of ^99m^Tc-IDA-D-[c(RGDfK)]_2_ and ^123^I-c(RGDyV). The SPECT/CT images of an atherosclerotic mouse 30 min after the intravenous injection of (**A**) ^99m^Tc-IDA-D-[c(RGDfK)]_2_ and (**B**) ^123^I-c(RGDyV). The representative coronal, sagittal and transverse planar images are shown. The aortic arch region show preferential accumulation for ^99m^Tc-IDA-D-[c(RGDfK)]_2_ (2.8%ID/g; arrows), compared to ^123^I-c(RGDyV) (1.1%ID/g; arrowheads). The CT images were acquired at 10 min post-contrast injection and the SPECT images were acquired during 30 min at 30 min post-injection of the radiolabeled probe. %ID/g, percentage injected dose per gram of tissue.

Having shown better sensitivity of RGD dimer peptide to detect the extent of atherosclerotic lesion in aortic tissues, we performed SPECT/CT imaging in the atherosclerotic model to evaluate whether ^99m^Tc-IDA-D-[c(RGDfK)]_2_ imaging was superior to the representative monomeric RGD-based radiotracer ^123^I-c(RGDyV) imaging (Fig. 4). At 30 min post-injection in the subsiding blood activity, atherosclerotic lesions were best visualized by ^99m^Tc-IDA-D-[c(RGDfK)]_2_ (Fig. 4A) and by ^123^I-c(RGDyV) (Fig. 4B). We observed a more intense signal by ^99m^Tc-IDA-D-[c(RGDfK)]_2_ in the aortic arch lesions compared to the signal by ^123^I-c(RGDyV), which was imaged in the same mouse on consecutive days. The signal intensity of ^99m^Tc-IDA-D-[c(RGDfK)]_2_ in the atherosclerotic arteries was more significant than the signal intensity of ^123^I-c(RGDyV) (2.8%ID/g versus 1.1%ID/g). Imaging by ^99m^Tc-IDA-D-[c(RGDfK)]_2_ showed overall improved targeting ability to detect the extent of plaque development.

### QD605-D-[c(RGDfK)]_2_ and QD605-c(RGDyK) Uptake by Tissue-based Assay

**Figure 5:**
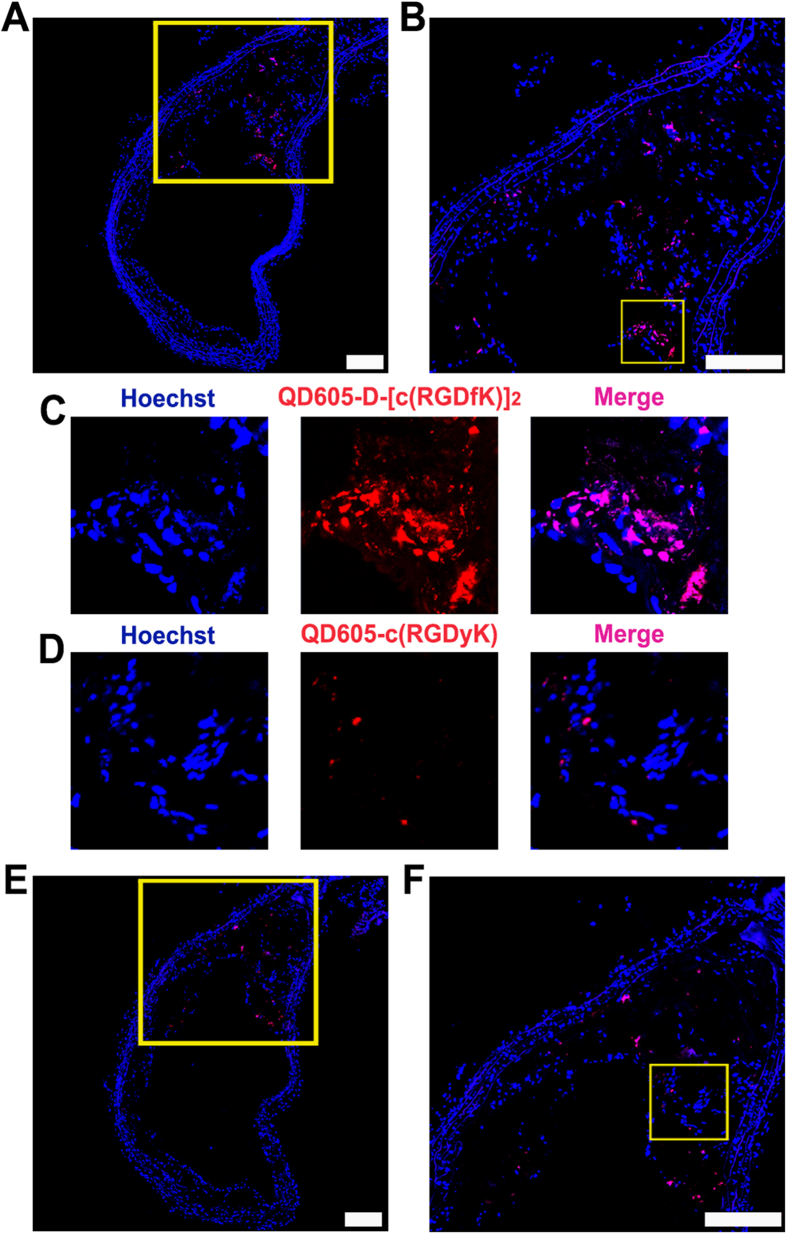
Comparison of plaque binding using a fluorophore-conjugated [c(RGDfK)]_2_ and c(RGDyK) for tissue-based assay. **A**–**C**, The confocal microscopic images of a cross-section of a high-risk atherosclerotic plaque after incubation with QD605-D-[c(RGDfK)]_2_. **D**–**F**, Confocal microscopic images of the adjacent section after incubation with QD605-c(RGDyK). **B** and **F**, The magnified images of the rectangle in **A** and **E**, respectively. **C** and **D,** High-power views, which correspond to the boxes in images **B** and **F**, respectively, show significant difference in probe uptake and prove the better sensitivity of QD605-D-[c(RGDfK)]_2._ Thirty minutes after the probe incubation, all fluorescence images were obtained with identical exposure times. Scale bars, 200 μm.

To verify *in vivo* SPECT/CT imaging data (Fig. 4), we next investigated the use of a fluorescently labeled RGD-dimer peptide, QD605-D-[c(RGDfK)]_2_, for more sensitive targeting of atherosclerotic plaques compared to a fluorophore-conjugated RGD-monomer peptide (i.e., QD605-c(RGDyK)) in the tissue-based assay. To compare the RGD dimer and monomer peptides’ uptake in aortic tissue sections, we conjugated fluorescent quantum dots (QD605, emission approximately 605 nm) to each peptide and used them to stain the plaque cryosections. Using confocal fluorescence microscopy, we readily identified the binding signal in consecutive sections. This study showed a substantially elevated uptake of QD605-D-[c(RGDfK)]_2_, compared to QD605-c(RGDyK), in the region corresponding to the gross location of atherosclerotic lesion, as Figs 5A–F show. Rapid and effective visualization of high-risk atherosclerotic plaque burden in the aorta sections by fluorescence microscopy indicated better sensitivity and selectivity of QD605-D-[c(RGDfK)]_2_ over QD605-c(RGDyK) and the potential to apply in clinical pathology analysis.

## Discussion

Most acute vascular events result from sudden luminal thrombosis due to rupture of an atherosclerotic plaque. Preventing such complications of atherosclerosis is the most urgent need to improve the survival of patients with cardiovascular disease. Contrast-enhanced x-ray angiography, which is the gold standard imaging tool used in clinics, only identifies luminal anatomy and rarely captures arterial wall characteristics, although the association between plaque composition and lesion instability has become obvious^5^. Thus, accurate discrimination between stable and vulnerable plaques remains a clinical challenge^5^,^7^,^8^,^13^,^14^.

In the present study, we demonstrated the feasibility of SPECT/CT imaging with α_v_β_3_ integrin-targeted ^99m^Tc-IDA-D-[c(RGDfK)]_2_ for specific detection of rupture-prone high-risk atherosclerotic plaques in a mouse model of atherosclerosis. The use of ^99m^Tc-IDA-D-[c(RGDfK)]_2_ was based on the premise that neovascularization is deeply associated with atheroma disruption or erosion, and thus the expression of integrin α_v_β_3_ by angiogenic endothelial cells can provide an important target for atherosclerosis staging. Imaging by SPECT/CT showed focal increases in the ^99m^Tc-IDA-D-[c(RGDfK)]_2_ signal in advanced lipid-rich plaques inside a mouse aorta, compared to the significantly low signal in normal areas of the same aorta or in the aortas of wild-type control mice (2.98 ± 0.64%ID/g versus 0.41 ± 0.10%ID/g for atherosclerotic and normal aortas, respectively; *P* < 0.001). Autoradiography and histopathology corroborated the *in vivo* data by revealing specific vulnerable plaque characteristics such as a large necrotic core and a thin fibrous cap.

The biological insights and experimental knowledge in understanding key processes of atherosclerosis that contribute to a lesion’s initiation, progression and complication have advanced markedly^9^,^10^,^11^,^12^. This has spurred many efforts to develop molecular imaging strategies to identify destabilized atherosclerotic plaques that are likely provoke the onset of acute thrombotic events^13^,^14^,^16^. The cyclic peptide RGD is perhaps the best known ligand for targeting angiogenesis through its specific binding affinity for integrin α_v_β_3_ and it has been widely used as a cancer diagnostic agent^21^,^29^,^30^. An increasing number of reports have recently indicated that angiogenesis is a very pertinent hallmark that can be used for staging atherosclerosis^17^,^18^,^19^. Shown in this study demonstrates successful application of RGD peptides to sensitively detect neoangiogenesis in high-risk lesions for clinical SPECT/CT imaging.

Focus of this study was particularly given to comparing monomeric and dimeric RGD-based tracers for gauging atherosclerosis. A direct comparison of ^99m^Tc-IDA-D-[c(RGDfK)]_2_ with RGD monomer based ^123^I-c(RGDyV) indicate that ^99m^Tc-IDA-D-[c(RGDfK)]_2_ has better *in vivo* targeting with a 3.7-fold higher affinity for unstable atherosclerotic lesion. *In vitro* tissue assay using QD605-D-[c(RGDfK)]_2_ and QD605-c(RGDyK) also showed superior binding property of the RGD dimer-based probe. Different fluorophore labeling using carboxyfluorescein and intensity quantification showed identical results (Supplementary Figure 4), proving such sensitivity difference arise from intrinsic property of RGD derivatives not fluorophore conjugation. Enhanced specific targeting may be due to improved avidity to integrin α_v_β_3_ of dimeric tracer over monomeric form as the interaction between integrins and their physiologic binding partners in the extracellular matrix involves multivalent binding sites with clustering of integrins.

Of note, the approach showcased in this study can be readily translated into the clinic, where its ultimate utility can be assessed. The noninvasive nuclear imaging technique SPECT has high sensitivity and quantification ability. The radionuclide ^99m^Tc can be obtained by daily elution from the ^99^Mo/^99^^m^Tc-generator, and is thus convenient and suitable for routine clinical use. Furthermore, *in vivo* imaging showed sufficient signal intensity for delineating aortic lesions and superior imaging performance compared with other monomer RGD based strategies. As expected, based on our previous study^31^, the mean uptake of ^99m^Tc-IDA-D-[c(RGDfK)]_2_ in atherosclerotic plaques (2.98 ± 0.34%ID/g) was several folds lower (because of volume difference) than the uptake reported in α_v_β_3_ integrin-expressing tumors (12.4 ± 3.89%ID/g)^31^. Despite the small dimension of atherosclerotic lesions, our results suggest that visualization of high-risk plaques in human artery may be possible with α_v_β_3_ integrin-specific SPECT/CT imaging. It must be highlighted that ^99m^Tc-IDA-D-[c(RGDfK)]_2_ depicts minimum background signal in chest SPECT/CT, which is especially beneficial for coronary artery imaging. By contrast, atherosclerosis imaging with ^18^F-FDG PET has been suffered with great background myocardial uptake because of glucose consumption by the heart muscle itself^36^. In addition, ^99m^Tc-IDA-D-[c(RGDfK)]_2_ SPECT/CT may have an advantage over previous preclinical MRI studies for complete targeting of intraplaque microvessels because of the small probe size compared to MRI contrast agents, α_v_β_3_ integrin-specific nanoparticles^25^,^26^.

To reach beyond the tools available in laboratory research, generalized, large, prospective clinical trials are needed to confirm the illustrated results of preclinical small animal imaging with ^99m^Tc-IDA-D-[c(RGDfK)]_2_ SPECT/CT. Typical atherosclerotic plaque regions that can be imaged in mouse models only include the larger vessels such as the abdominal aorta, the carotid arteries, the aortic arch, and the aortic root, as displayed in this report. It is challenging, but there is great interest in directly imaging thrombosis-prone plaques in small coronary arteries, which commonly cause acute myocardial infarction. Therefore, ^99m^Tc-IDA-D-[c(RGDfK)]_2_ SPECT/CT needs to be evaluated for assessing the likelihood of atherosclerotic events in more generalized clinical settings. Potential for effective therapeutic monitoring tool is another valuable investigation strategy to facilitate clinical use of ^99m^Tc-IDA-D-[c(RGDfK)]_2_. Treatment efficacy by clinically available drug such as statin can be directly evaluated using ^99m^Tc-IDA-D-[c(RGDfK)]_2_ SPECT/CT of patients.

In conclusion, as a marker of plaque vulnerability, the ^99m^Tc-IDA-D-[c(RGDfK)]_2_ SPECT signal can help identify patients with the highest risk of cardiovascular events. In addition, ^99m^Tc-IDA-D-[c(RGDfK)]_2_ SPECT/CT has the potential to be used successfully as a surrogate tool to monitor clinical interventions and antiatherosclerotic therapies aimed at mitigating cardiovascular events. Based on promising animal imaging results, we anticipate its clinical translation in the coming years.

## Methods

### Chemistry and Radiochemistry

All commercial reagents and solvents were purchased from Sigma-Aldrich (St. Louis, MO, USA) and used without further purification, unless otherwise specified. The precursors (i.e., IDA-D-[c(RGDfK)]_2_ and IDA-D-[c(RADfK)]_2_) and three radiotracers (i.e., ^99m^Tc-IDA-D-[c(RGDfK)]_2_, ^99m^Tc-IDA-D-[c(RADfK)]_2_, and ^123^I-c(RGDyV)) were prepared in accordance with previously described methods^31^,^32^. ^99m^Tc-pertachnate was eluted on a daily basis from ^99^Mo/^99m^Tc-generator (Samyoung Unitech, Seoul, Korea) and the radionuclide iodine-123 (Na^123^I) was purchased from Korea Cancer Center Hospital (Seoul, Korea).

In fluorescence tissue-based assay, to compare the plaque binding ability of the cyclic RGD dimer peptide ([c(RGDfK)]_2_) with cyclic RGD monomer peptide (c(RGDyK)), the molecule NH_2_-D-[c(RGDfK)]_2_ and NH_2_-c(RGDyK) was conjugated to fluorescent quantum dots (emission, approximately 605 nm; Qdot 605 ITK; Life Technologies, Carlsbad, CA, USA) using *N*-ethyl-*N*’-dimethylaminopropyl-carbodiimide chemistry in borate buffer.

### Mouse Model of Atherosclerosis

All animal experiments were carried out in accordance with the approved guidelines by the Seoul National University Bundang Hospital Animal Care and Use Committee. Apolipoprotein E knock out mice (male, n = 10) and C57BL/6J mice (male, n = 3) were purchased from the Jackson Laboratory (Bar Harbor, Maine, USA) at 8–10 weeks of age. The *ApoE-/-* mice were maintained on a high-cholesterol diet (0.2% total cholesterol, Harlan Laboratories, Indianapolis, IN, USA) for 40 weeks. At the time of imaging experiment, the mice had substantial atherosclerotic lesion growth resembling vulnerable plaques in humans. Wild-type mice with the C57BL/6J genetic background had been maintained on a regular diet and were used for the disease controls.

### Animal SPECT/CT Imaging and Analysis

We anesthetized mice with 2% isoflurane gas anesthesia. The *ApoE-/-* mice were then administered intravenously ^99m^Tc-IDA-D-[c(RGDfK)]_2_ (n = 4), ^99m^Tc-IDA-D-[c(RADfK)]_2_ (n = 3), ^123^I-c(RGDyV) (n = 1), or ^99m^Tc-pertachnate (n = 2) (each 37 MBq in 0.3 mL of saline) and C57BL/6J was administered ^99m^Tc-IDA-D-[c(RGDfK)]_2_ (n = 3). The mice were placed supine on the bed of an animal SPECT/CT scanner (NanoSPECT/CT, Bioscan Inc., Washington DC, USA). At 30 min post-injection, a high-resolution static scan of chest region was acquired in helical scanning mode in 24 projections during a 30 min period using a four-head scanner with 4 × 9 (1.4 mm) pinhole collimators. The energy window was set at 140 keV ± 15%. The SPECT imaging was followed by CT image acquisition with the animal in the same position. The CT images were obtained with the x-ray source set at 45 kVp and 177 μA after the injection of vascular contrast agent Fenestra VC (MediLumine Inc., Montreal, QC, Canada) to demonstrate the ability to visualize the vasculature with the CT scanner for correlation to a SPECT studies (Figs 1D–I and 2A–C). Following intravenous injection of 10 μl/g of Fenestra VC, CT was performed at a mean time of 10 minutes post-injection with acquisition time 270 s per CT scan. The analysis software HiSPECT (Version 1.0, Bioscan Inc., Poway, CA, USA) and InVivoScope (Version 1.43, Bioscan Inc., Poway, CA, USA) were used for image reconstruction and quantification, respectively. The SPECT images were reconstructed to produce an image size of 176 × 176 × 136 voxels with a voxel size of 0.2 × 0.2 × 0.2 mm. The CT images were 48 μm resolution acquisition with a voxel-pixel size of 0.20 : 0.192 mm. Details for image reconstruction and processing have been reported elsewhere^31^. Manually drawn two-dimensional regions of interest (ROIs) or three-dimensional volumes of interest (VOIs) were used to determine the accumulated radioactivity in units of %ID/g (with decay corrected to the time of injection) using 37–55.5 MBq radioactivity of ^99m^Tc as the reference source.

### Statistical Analysis

Comparisons between regions were performed using SPSS Statistics 19 (IBM, Armonk, NY, USA). All data were analyzed using SigmaStat software, version 3.5 (Systat Software, San Jose, CA, USA). Differences with a *P* value less than 0.001 were considered significant.

### Autoradiography

After performing animal SPECT/CT imaging with ^99m^Tc-IDA-D-[c(RGDfK)]_2_ and the negative-control peptide ^99m^Tc-IDA-D-[c(RADfK)]_2_, we dissected the aortic tissues of the atherosclerotic animals and laid these specimens flat on a phosphor imager (Fuji BAS-5000; Fujifilm Life Sciences, Stamford, USA) for 24 h. The generated autoradiographs were analyzed using a computer-based image analysis system (Multi Gauge software, Fujifilm Life Sciences). The specific uptake was expressed as photostimulated luminescence per millimeter squared (PSL/mm^2^).

### Histological Evaluation

To confirm the development of typical lipid-rich atherosclerotic plaques, the excised aortas were imaged intact. They then underwent Oil-Red-O staining. All photographic images were obtained by a digital camera.

To evaluate noninvasive imaging of atherosclerotic lesions, histopathology was performed on *in vivo* imaged aortas with an intense ^99m^Tc-IDA-D-[c(RGDfK)]_2_ signal. After undergoing SPECT/CT and autoradiography imaging, the excised aortas were fixed with 10% formalin, embedded in paraffin, cut into 5-μm sections and deparaffinized. The sections were subsequently stained with hematoxylin and eosin or Masson’s trichrome stain to characterize the morphology and composition of the recorded peak signal of the aorta. Bright field color micrographs were obtained on a BX51 microscope equipped with DP71 camera (Olympus Optical Co., Ltd., Tokyo, Japan).

### *In Vitro* Fluorescent Staining Assay in Plaque Tissue

Aorta tissues were dissected from apoE transgenic (*ApoE-/-*) mice (which were fed a high-cholesterol diet for 40 weeks) and wild-type C57BL/6J mice. The resected aortas were embedded in a tissue-freezing medium (Triangle Biomedical Sciences, Durham, NC, USA), frozen, and consecutively cryosectioned in 10-μm segments using a Cryocut Microtome (CM3050S, Leica, Solms, Germany). The tissue sections were thaw-mounted onto silane-coated microscope slides (Muto Pure Chemicals co., Tokyo, Japan), dried in an aeration room, and stored at –80 °C until use. To confirm *in vivo* data and to compare plaque tissue binding ability, we performed fluorescence staining with QD605-D-[c(RGDfK)]_2_ and QD605-c(RGDyK) on consecutive sections by the following steps: the 10-μm sections were washed with phosphate-buffered saline (PBS) and incubated with either QD605-D-[c(RGDfK)]_2_ or QD605-c(RGDyK) (10 pmol of peptide, 4 μg of QD605) for 30 min. The slides were then washed with PBS several times, counterstained with Hoechst33342 and mounted with Prolong Gold Antifade Reagent (Life Technologies, Carlsbad, CA, USA). The fluorescence images were captured with a confocal microscope (TCS NT4D, Leica, Solms, Germany) to identify the binding difference between the two agents.

## Additional Information

**How to cite this article**: Yoo, J. S. *et al.* SPECT/CT Imaging of High-Risk Atherosclerotic Plaques using Integrin-Binding RGD Dimer Peptides. *Sci. Rep.* **5**, 11752; doi: 10.1038/srep11752 (2015).

## Supplementary Material

Supplementary Information
