# Supplementary material for: SPECT/CT Imaging of High-Risk Atherosclerotic Plaques using Integrin-Binding RGD Dimer Peptides
Source: Sci Rep. 2015 Jun 30;5:11752. doi: 10.1038/srep11752 (PMC4485237; doi:10.1038/srep11752)
Supplement: Supplementary Information [file srep11752-s1.doc]

**SPECT/CT Imaging of High-Risk Atherosclerotic Plaques using Integrin-Binding RGD Dimer Peptides**

Jung Sun Yoo^2,3*^, Jonghwan Lee^4,5*^, Jae Ho Jung^1^, Byung Seok Moon^1^, Soonhag Kim^4,5+^, Byung Chul Lee^1,3+^, Sang Eun Kim^1,2,3+^

^1^Department of Nuclear Medicine, Seoul National University Bundang Hospital, Seoul National University College of Medicine, Seongnam, Republic of Korea, ^2^Smart Humanity Convergence Center, Program in Biomedical Radiation Sciences, Department of Transdisciplinary Studies, Graduate School of Convergence Science and Technology, Seoul National University, Suwon 443-270, Republic of Korea, ^3^Center for Nanomolecular Imaging and Innovative Drug Development, Advanced Institutes of Convergence Technology, Suwon 443-270, Republic of Korea, ^4^Institute for Bio-Medical Convergence, College of Medicine, Catholic Kwandong University, Gangneung 210-701, Republic of Korea, ^5^Catholic Kwandong University International St. Mary’s Hospital, Incheon 404-834, Republic of Korea

*These authors contributed equally to this study.

^+^Correspondence and requests for materials should be addressed to B.C.L. ([leebc@snu.ac.kr](mailto:leebc@snu.ac.kr)) or S.K. ([kimsoonhag@empal.com](mailto:kimsoonhag@empal.com)) or S.E.K. ([kse@snu.ac.kr](mailto:kse@snu.ac.kr))

**LIST OF SUPPLEMENTARY ITEMS**

**Supplementary Figure 1:** Immunofluorescent assessment of a fluorophore-conjugated [c(RGDfK)]_2_ targeting of atherosclerotic plaque

**Supplementary Figure 2:** Quantification of *In Vitro* Peptide Binding in Plaque Tissue

**Supplementary Figure 3:** Characterization of FAM-Asp[c(RGDfK)]_2_ using HPLC analysis and mass spectrometry

**Supplementary Figure 4:** Characterization of c[RGDfK(FAM)]) using HPLC analysis and mass spectrometry

**Supplementary Methods**

**Supplementary Figure 1.** Immunofluorescent assessment of a fluorophore-conjugated [c(RGDfK)]_2_ targeting of atherosclerotic plaque. Shown are frozen aorta serial sections stained with FAM-Asp[c(RGDfK)]_2_ (**A, B**) or antibodies (red) to Integrin α_v_ (**E**, activated endothelial cell), Integrin β_3_ (**C**, activated endothelial cell), CD31 (**F**, endothelial cell), and CD68 (**D**, macrophage). All sections were counterstained with Hoechst33342 (blue) to identify nuclei. The first image (**A**) shows a whole aorta section image and the others (**B-F**) depicts representative magnified images of the plaque corresponding to the rectangle in (A). The autofluorescence signal (an asterisk in **B**) from elastic fibers of endothelium of the aorta enables identification of plaque location and tissue orientation.


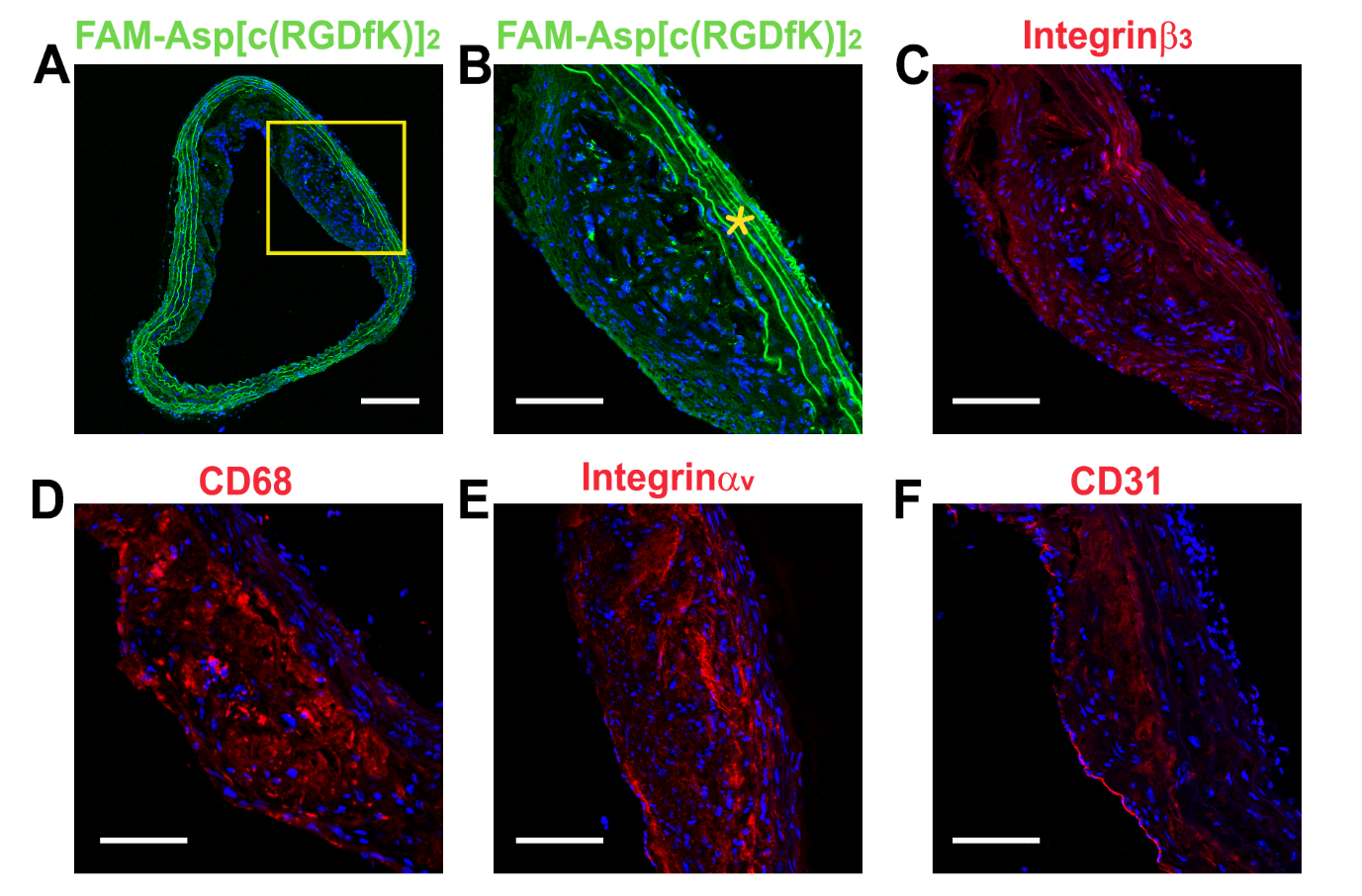


**Supplementary Figure 2.** Quantification of *In Vitro* Peptide Binding in Plaque Tissue. Quantitative intensity analysis was performed with three separate images of fluorescent stained plaque sections for FAM-Asp[c(RGDfK)]_2_ and c[RGDfK(FAM)]). Data are presented as mean ± SD.


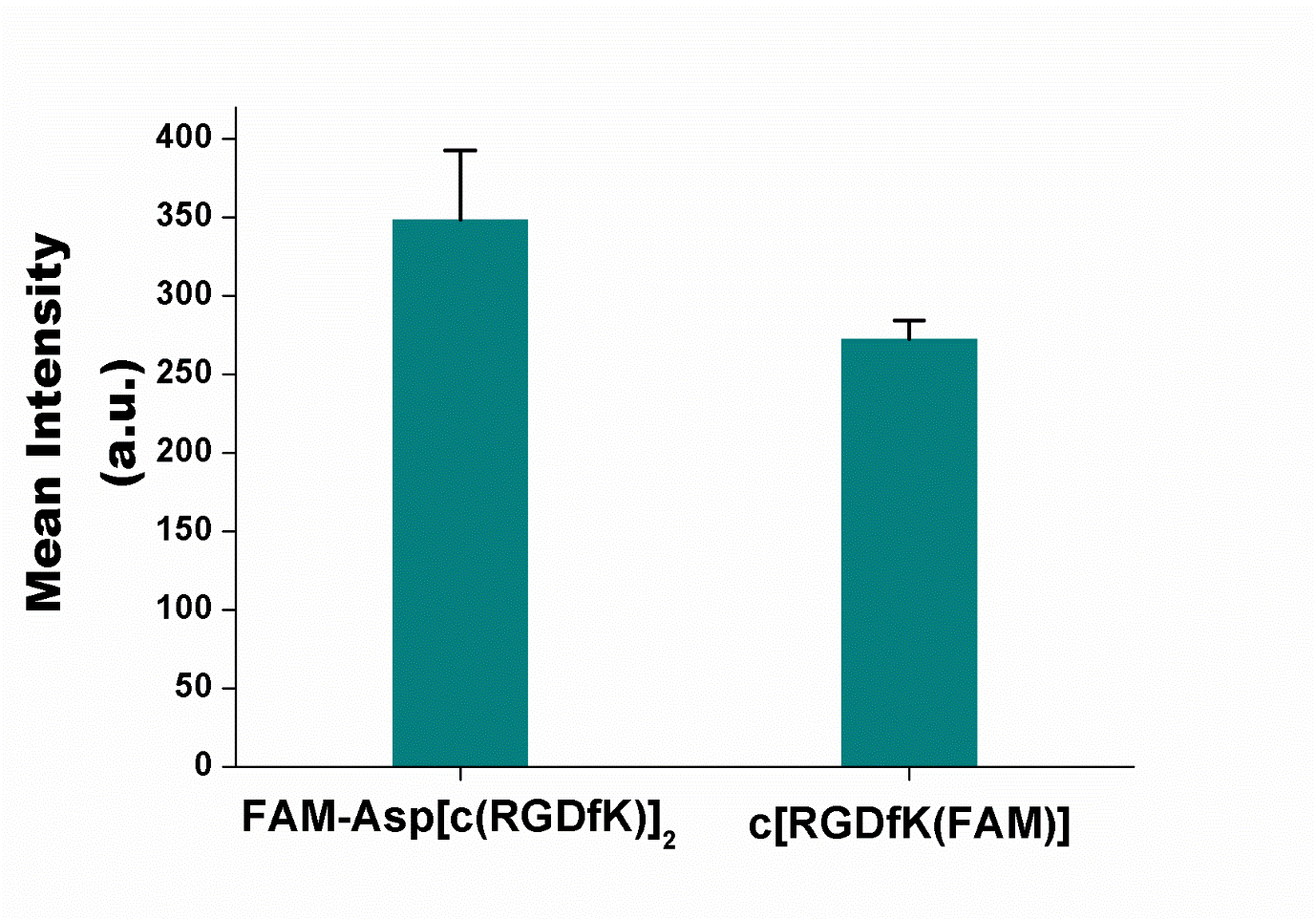


**Supplementary Figure 3.** Characterization of FAM-Asp[c(RGDfK)]_2_ using HPLC analysis and mass spectrometry. HPLC analysis shows more than 95% purity (A) and LC-MS spectrum confirms successful synthesis.


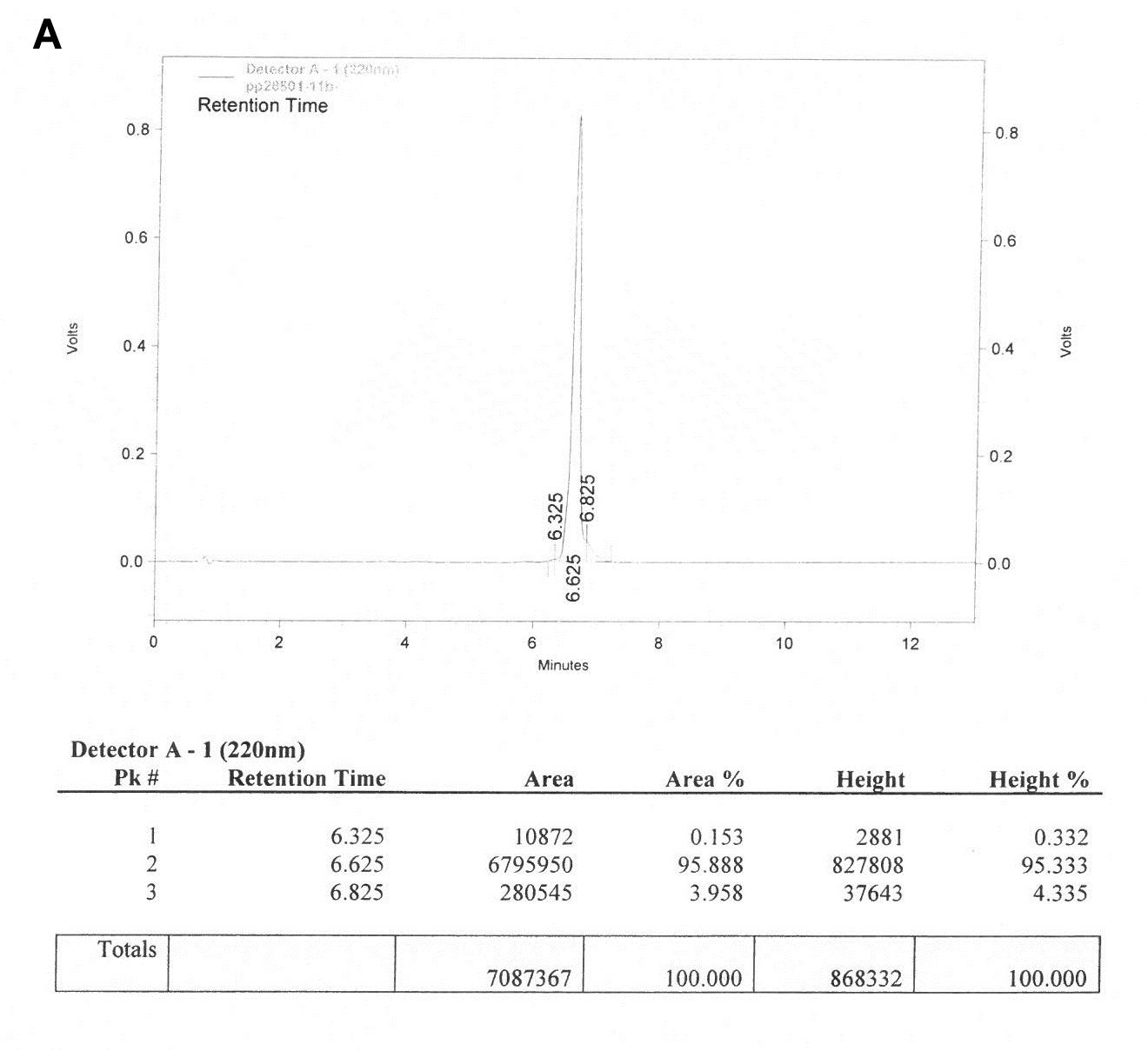


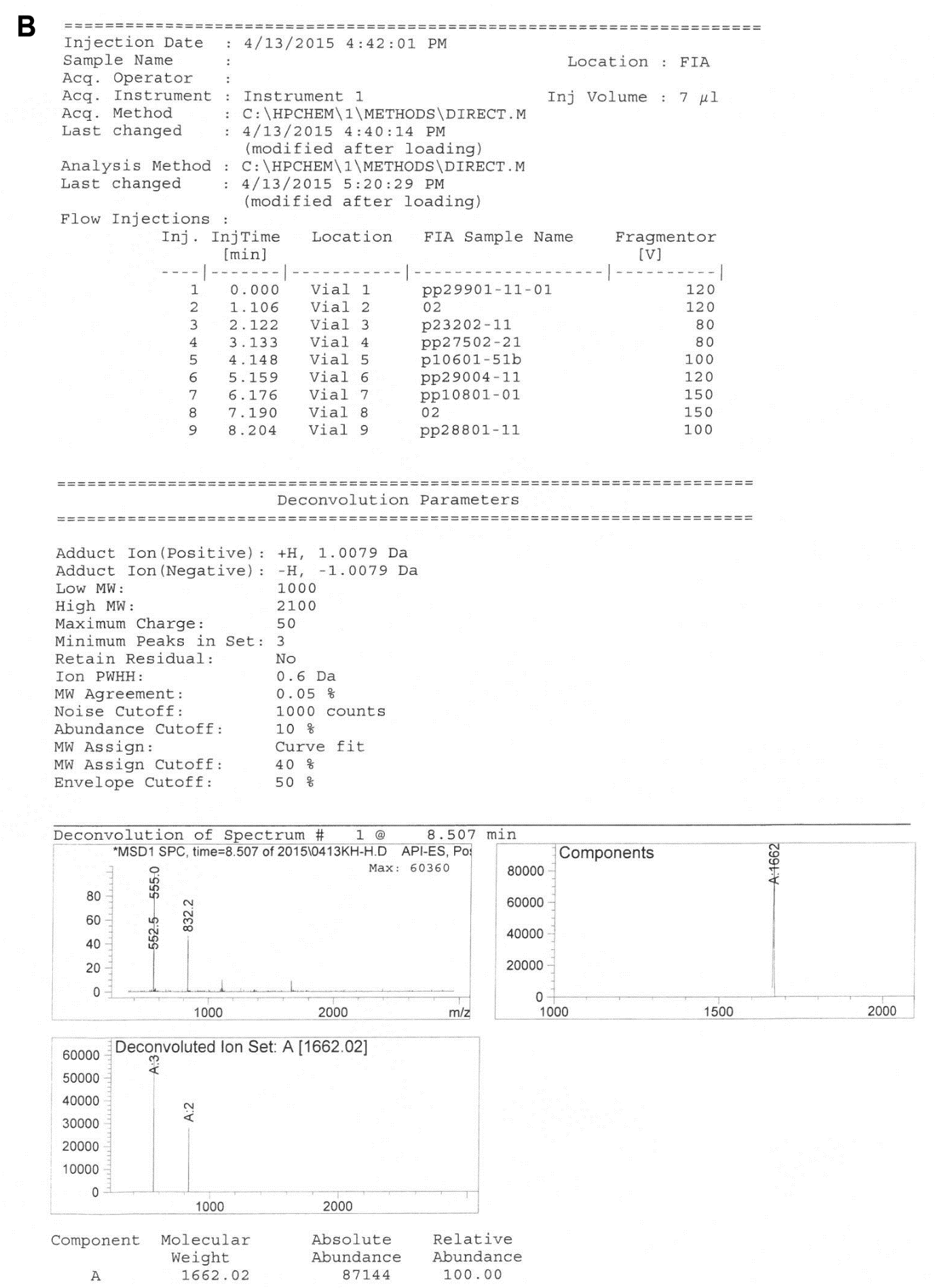


**Supplementary Figure 4.** Characterization of c[RGDfK(FAM)]) using HPLC analysis and mass spectrometry. HPLC analysis shows more than 95% purity (A) and LC-MS spectrum confirms successful synthesis.


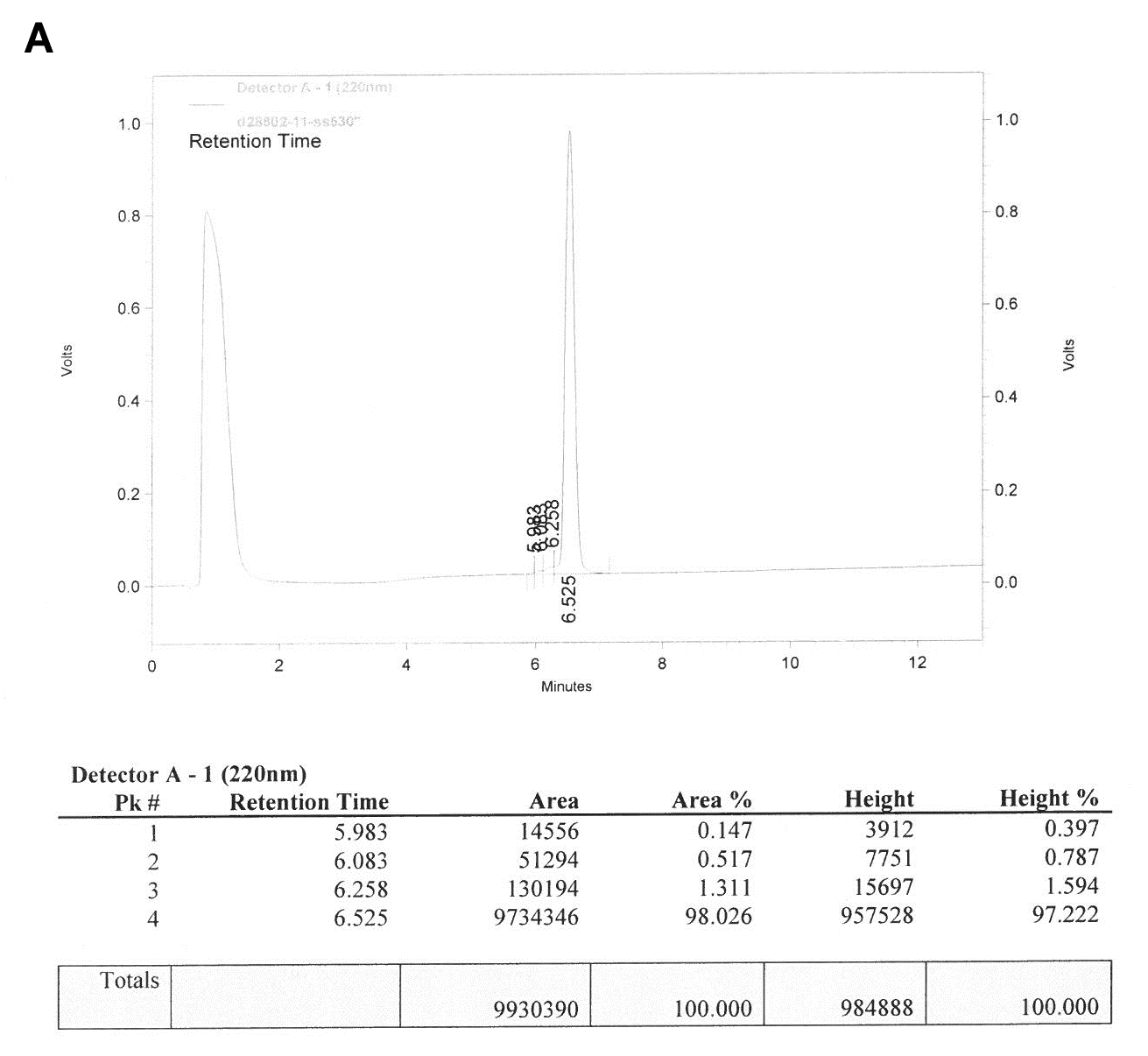


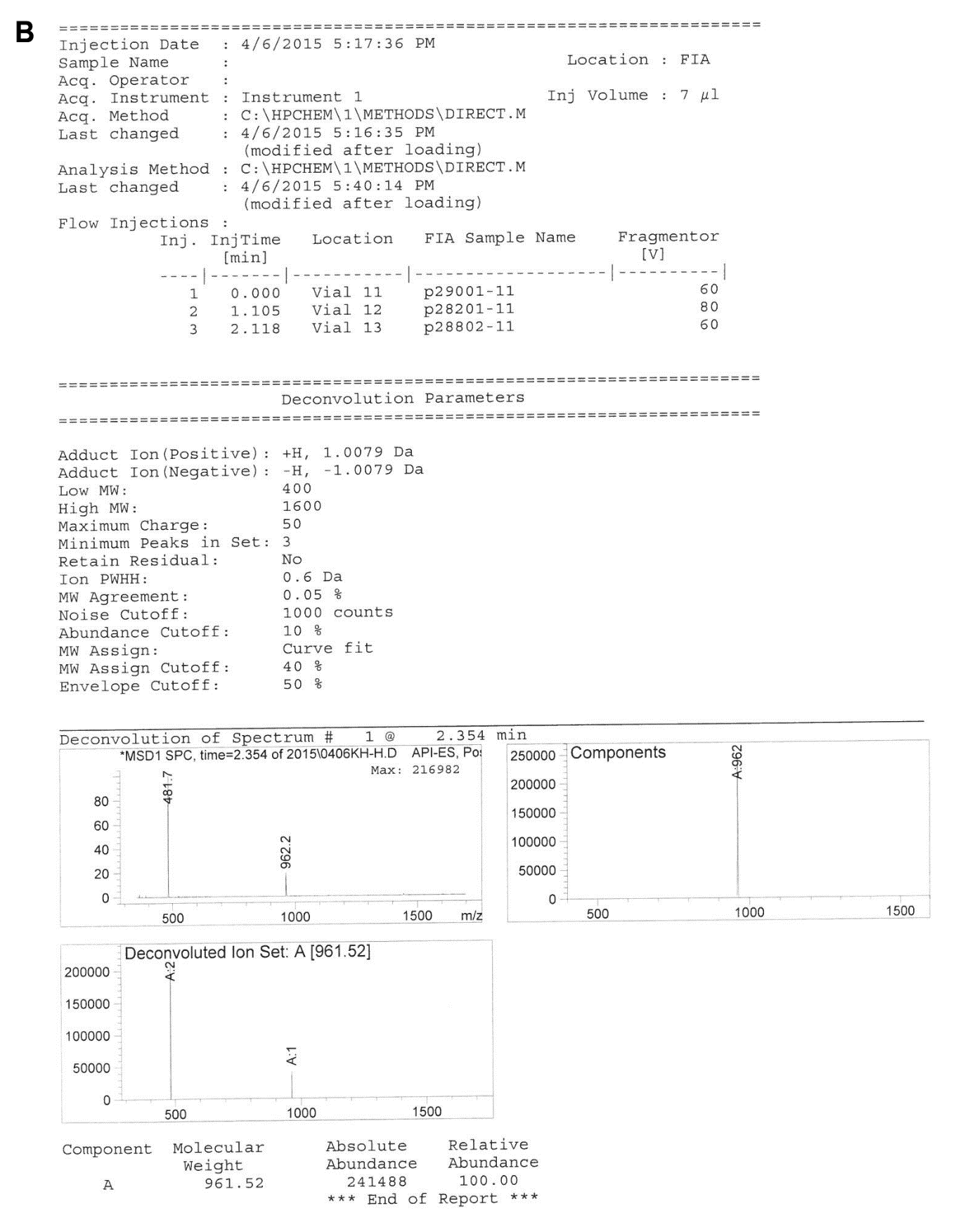


**Supplementary Methods**

**Peptide Synthesis and Fluorophore Labeling**

RGD dimer and monomer peptides were either synthesized in our laboratory using standard protocols for fluorenylmethoxycarbonyl (Fmoc) solid-phase synthesis or made by Peptron (Daejeon, Republic of Korea). Then, the peptides were conjugated to the Fluorescein-5(6)-carbonyl group (FAM, Peptron) using N-hydroxysuccinimide ester chemistry in dimethyl sulfoxide. FAM labeled peptides (FAM-Asp[c(RGDfK)]_2_, c[RGDfK(FAM)]) were purified to >95% purity using C-4 (dimer) or C-18 (monomer) reverse phase high-performance liquid chromatography (HPLC, Shimadzu Prominence, Kyoto, Japan) with sequential changes of acetonitrile gradient in 0.1 % trifluoroacetic acid and confirmed by mass spectrometry (HP/Agilent 1100 series LC/MSD, Santa Clara, CA, USA).

**Immunofluorescence Staining**

Immunofluorescence staining was performed using serial sections of the aorta which underwent SPECT/CT imaging. Briefly, the excised aorta was embedded in a tissue-freezing medium (Triangle Biomedical Sciences, Durham, NC, USA), frozen, and consecutively cryosectioned in 10-µm segments using a Cryocut Microtome (CM3050S, Leica, Solms, Germany). The tissue sections were thaw-mounted onto silane-coated microscope slides (Muto Pure Chemicals co., Tokyo, Japan), dried in an aeration room, and stored at -80 °C until use. The tissue sections were rinsed with phosphate-buffered saline (PBS) and fixed with 4 % paraformaldehyde for 20 minutes at room temperature (RT). After an additional series of washed with PBS, they were cleared 3 % sodium deoxycholate solution for 2 hours at RT, blocked with 20 % normal goat serum in 1 % BSA-PBS for 2 h at 37 °C, incubated with the primary antibodies at 4 °C overnight (> 17 hours). Specific antibodies against Integrin α_v_ (1:100, BD, 611013), Integrin β_3_ (1:100, Abcam, ab75872), endothelial cell marker CD31 (1:100, BD, 553370), and macrophage marker CD68 (1:150, Abd serotec, MCA1957) were used on atherosclerotic sections as indicated. Subsequently, the slides were rinsed three times with PBS and incubated with secondary antibodies (1:400, Alexa Fluor 594 labeled, Life technologies, Carlsbad, CA, USA) at 4 °C for 2 hours. Then, they were washed with PBS several times, counterstained with Hoechst33342 (1:750, Life Technologies), and mounted with Prolong Gold antifade reagent (Life Technologies). The adjacent section was treated with FAM conjugates of RGD dimer peptide to identify co-localization with antibody positive signals. The images were captured with a confocal microscope (A1, Leica, Solms, Germany).

**Quantitative Image Analysis of *In Vitro* Peptide Binding Assay**

To quantify *in vitro* binding of FAM-Asp[c(RGDfK)]_2_ and c[RGDfK(FAM)], cryo-sections of the atherosclerotic aorta were incubated with each peptide (10 μM, 30 minutes, RT). The fluorescence microscopic images at three different sites of the aortic plaque were taken using a confocal microscope (A1, Leica, Solms, Germany) with identical measurement conditions. The images were further optimized for brightness and contrast then the adjusted settings were applied to all other images. The mean intensity of peptide positive signal for each image was calculated by ImageJ. The standard deviation (SD) for three different measurements of mean intensity was also calculated with ImageJ. Data are presented as mean ± SD.
